# Development of a Surface Coating Technique with Predictive Value for Bead Coating in the Manufacturing of Amorphous Solid Dispersions

**DOI:** 10.3390/pharmaceutics12090878

**Published:** 2020-09-15

**Authors:** Eline Boel, Piyush Panini, Guy Van den Mooter

**Affiliations:** Department of Pharmaceutical and Pharmacological Sciences, Drug Delivery and Disposition, KU Leuven, 3000 Leuven, Belgium; eline.boel@kuleuven.be (E.B.); piyush.panini@kuleuven.be (P.P.)

**Keywords:** bead coating, spray drying, film casting, surface coating

## Abstract

The aim of this paper was to investigate whether a surface coating technique could be developed that can predict the phase behavior of amorphous solid dispersions (ASDs) coated on beads. ASDs of miconazole (MIC) and poly(vinylpyrrolidone-*co*-vinyl acetate) (PVP-VA) in methanol (MeOH) were studied as a model system. First, the low crystallization tendency of the model drug in MeOH was evaluated and confirmed. In a next step, a drug loading screening was performed on casted films and coated beads in order to define the highest possible MIC loading that still results in a one-phase amorphous system. These results indicate that film casting is not suitable for phase behavior predictions of ASDs coated on beads. Therefore, a setup for coating a solid surface was established inside the drying chamber of a spray dryer and it was found that this surface coating technique could predict the phase behavior of MIC-PVP-VA systems coated on beads, in case an intermittent spraying procedure is applied. Finally, spray drying was also evaluated for its ability to manufacture high drug-loaded ASDs. The highest possible drug loadings that still result in a one-phase amorphous system were obtained for bead coating and its predictive intermittent surface coating technique, followed by spray drying and finally by film casting and the continuous surface coating technique, thereby underlining the importance for further research into the underexplored bead coating process.

## 1. Introduction

Amorphous solid dispersions (ASDs) are one of the most effective approaches to enhance the oral bioavailability of drug compounds suffering from low aqueous solubility [1]. With this formulation strategy, the poorly water-soluble drug is molecularly dispersed within an inert polymer matrix (“carrier”) in the solid state [2]. Despite the considerable expertise, acquired over the last couple of decades, few ASDs have been commercialized thus far [3]. This is mainly due to physical stability issues that may occur during processing as well as during storage, implying amorphous demixing (i.e., drug-rich and polymer-rich phase) and/or crystallization of the molecularly dispersed drug, ultimately leading to loss of the solubility/dissolution rate advantage [2,4]. Continuous research efforts within this field are hence paramount to bridge the gap between the increasing number of new drug candidates possessing unfavorable physicochemical properties on the one hand, and physical stability issues entailing limited commercialization on the other hand.

ASDs can be prepared by the rapid cooling of a melt (i.e., heat-based methods), precipitation of a drug-carrier solution (i.e., solvent-based methods) or by direct solid conversion methods (i.e., mechanochemical activation based methods) [5]. Within the category of the solvent-based ASD manufacturing methods, the well-established spray drying process and the less explored bead coating or fluid-bed coating process are of interest, because the differences between them offer attractive prospects with a view to the physical stability discussion.

In spray drying, a drug–polymer solution is atomized through a nozzle, thereby generating small droplets which are quickly dried in the drying chamber. Due to the very fast solvent evaporation, the physical structure of the drug-polymer system will be kinetically trapped, rendering amorphous materials [6]. Although spray drying has proven its usefulness to industrially prepare ASDs, a major drawback is that this technique requires down-stream processing steps to obtain dosage forms (e.g., tablets) acceptable to be administered to patients [6]. In general, spray-dried powders are characterized by a low bulk density and poor flowability, requiring the implementation of a granulation step as pre-densification before final tablet compression. Mechanical and heat stresses during dry granulation can have negative implications for the physical stability of ASDs, as reported by Vaka et al., who observed a substantial reduction in bioavailability of the drug after roller compaction [7]. If wet granulation is applied, contact with solvents can lead to plasticization and hence physical stability issues because of increased molecular mobility [8]. Moreover, compression forces can imply amorphous demixing and crystallization and can thus also nullify the initial solubility advantage of the ASD [9,10,11].

In contrast, if bead coating is selected as the ASD manufacturing technique, potential detrimental down-stream processing steps are avoided, since the coated beads can be readily administered to patients via capsule formulations. Bead coating applies the principle of layering a drug-polymer solution on inert beads. More specifically, the drug-polymer solution is sprayed via a nozzle onto cores that are continuously subject to motion, provided by fluidization through an inlet air stream. The fluidization aspect of this process enables an in-device drying step to reduce the residual solvent level, thereby circumventing a transfer to a post-drying device, and thus further minimizing potential stability issues. The versatility of the device also allows for the subsequent application of an additional coating layer, either to serve as a drug release rate controlling membrane or as a protection barrier against surface crystallization [12]. In addition to process-related advantages, bead coating also offers interesting opportunities concerning clinical aspects. The resulting coated pellets formulation (i.e., multiparticulate dosage form) exhibits a better spread in the gastro-intestinal tract and thus involves a reduced risk of dose-dumping [13,14]. This ASD formulation also enables more flexible dose adjustments in the clinical phases of the drug development process, as compared to a single-unit tablet formulation, and can be considered an interesting choice to compose fixed-dose combinations [15].

Despite the successful development and marketing of Sporanox^®^, few research attempts thoroughly examined bead coating as an ASD manufacturing technique and scientific literature is very scarce. Moreover, the main focus in the current literature lies on simply reporting of the dissolution behavior and does not encompass a profound understanding of the physical chemistry behind the manufacturing process and formulation [13,16,17,18]. Longer processing times, as compared to spray drying, and the challenges often encountered in finding optimal parameter settings, so that agglomeration phenomena are avoided, might serve as a possible explanation. Within the pharmaceutical industry, film casting is often used as a screening test to assess drug–polymer miscibility. Nevertheless, this technique is not suitable for phase behavior predictions of ASDs manufactured with spray drying or bead coating, given the low rate of solvent evaporation. This research therefore aims to investigate whether and how a surface coating technique can be developed that can predict the phase behavior of the ASDs coated on beads, which forms an important first step towards a better understanding of the bead coating process. More specifically, solid dispersions of miconazole (MIC) and poly(vinylpyrrolidone-co-vinyl acetate) (PVP-VA) in methanol (MeOH) were studied as a model system. First, the low crystallization tendency of the model drug in MeOH was evaluated. In addition to the development of the surface coating technique and the assessment of its predictive value, the technique was also evaluated for its ability to manufacture high drug-loaded ASDs, in comparison to bead coating, spray drying and film casting. Since the focus lies on the development of the predictive technique and its ability to manufacture high drug-loaded ASDs at time point zero, physical stability studies were not within the aim of this study and therefore not conducted.

## 2. Materials and Methods

### 2.1. Materials

MIC was kindly donated by Janssen Pharmaceutica N.V. (Beerse, Belgium). Kollidon-VA 64 (PVP-VA; Mw 24–30 kDa) was obtained from BASF ChemTrade GmbH (Ludwigshafen, Germany), and ACROS (Geel, Belgium) supplied MeOH (purity ≥ 99.8%) and phosphorus pentoxide. Microcrystalline cellulose (MCC) beads (Vivapur^®^ 700: 18–25 mesh, 710–1000 µm) were purchased from JRS Pharma GmbH (Rosenberg, Germany).

### 2.2. Production of MCC Tablets

Pure MCC beads were milled with a laboratory cutter mill (Ika, Staufen, Germany) and sieved through a 300 µm sieve. The resulting MCC powder was compressed by use of a single punch manual tablet press RQPBA15 (Rodac International, Sittard, The Netherlands) with a die of 13 mm diameter. A pressure of 369.5 MPa was applied using a 30 s dwell time, resulting in 390 ± 15 mg MCC tablets with a thickness of 2 mm.

### 2.3. Surface Coating and Spray Drying

A Büchi mini spray dryer B-191 (Büchi, Flawil, Switzerland) was used for coating the surface of the MCC tablets, as well as for the production of spray dried powder. A glass cylinder was placed inside the drying chamber, which serves as a supporting plate for the tablet, while still allowing sufficient air flow for simultaneous production of spray dried powder that can be collected in the collection vessel. A more detailed description of the setup can be found in Section 3.3. Accurate amounts of MIC and PVP-VA were dissolved in 20.0 mL MeOH to obtain a solid content of 10% *w*/*v*. The drying air temperature in the drying chamber was set at 40 °C (corresponding to the bed temperature (T_bed_) during the bead coating procedure) and the drying air flow rate at 33 m³/h. The feed solution flow rate was set at 7 mL/min and the atomization air flow rate at 10 L/min. When the coating procedure was finished, an additional drying step of 5 min was implemented. Coated tablets and spray dried powder were further dried in a vacuum oven for 72 h at room temperature and afterwards, stored at −28 °C in the presence of phosphorus pentoxide until further analysis. Half of the coating of the MCC tablets was scraped off with a scalpel blade for modulated differential scanning calorimetry (mDSC) analysis (in triplicate), and the other half was analyzed as such with X-ray powder diffraction (XRPD). The spray dried powders were analyzed as such with both solid-state characterization techniques.

Evaluation of the crystallization tendency of MIC in MeOH was carried out with the same Büchi mini spray dryer. For this purpose, an accurate amount of MIC was dissolved in 20.0 mL MeOH (solid content: 2.3% *w*/*v*) and the following parameters were applied: a drying air temperature of 65 °C (corresponding to the boiling point of MeOH), drying air flow rate of 33 m³/h, a feed solution flow rate of 5 mL/min and an atomization air flow rate of 15 L/min. The samples were prepared in triplicate. The sticky spray dried outcomes were stored at ambient conditions and analyzed with mDSC right after spray drying (day 0), after one day (day 1) and after one week (day 7) of storage.

### 2.4. Film Casting

An accurate amount of MIC and PVP-VA was dissolved in MeOH, in order to obtain a solid content of 10% *w*/*v*. The solutions were cast on a glass plate coated with Teflon (High-tech-flon, Konstanz, Germany) and dried for 72 h at an ambient temperature. The solvent evaporation rate was controlled by covering the casted solutions with a funnel. All films were prepared in triplicate and analyzed as such with mDSC and XRPD.

### 2.5. Bead Coating

Coated beads were prepared using a Mini-Glatt fluid bed coater (Glatt, Binzen, Germany) in a bottom spray setup, equipped with a Würster insert. The partition height was set at 7.5 mm. A 10% *w*/*v* drug-polymer solution in MeOH with total solid content of 20.0 g was coated onto 150.0 g MCC beads. First, the MCC beads were fluidized for 45 min at 25 m³/h, with the inlet temperature (T_inlet_) set at 50 °C. After this preheating step, the drug-polymer solution was coated onto the beads at a feed rate of 1 mL/min, while applying an atomization air pressure of 1 bar. The T_inlet_ setting remained the same and ensures a T_bed_ of approximately 40 °C. During the coating procedure, the drying air flow was kept between 33 and 35 m³/h, and the tapping frequency was installed at 5 s. For the formulation with 50% MIC loading, the T_inlet_ and feed rate were lowered to 38 °C and 0.6 mL/min, respectively, because of agglomeration issues due to stickiness. Once the spraying was finished, a drying step of 5 min was implemented. The coated beads were unloaded and further dried in a vacuum oven for 72 h at room temperature. Afterwards, the beads were stored at −28 °C in the presence of phosphorus pentoxide until further analysis. The coated pellets were milled for 10 s using a laboratory cutter mill (Ika, Staufen, Germany) to obtain fine powder that allows optimal thermal contact during mDSC analysis. Potential milling-induced crystallinity was evaluated with XRPD.

### 2.6. Solid-State Characterization

#### 2.6.1. Modulated Differential Scanning Calorimetry (mDSC)

The phase behavior of the formulations was evaluated with a Discovery DSC 2500 (TA Instruments, Leatherhead, UK), equipped with a refrigerated cooling system (RCS 90) and a dry nitrogen purge with a flow rate of 50 mL/min. Calibration for temperature, enthalpy and heat capacity was carried out using indium and sapphire standards, respectively. The following parameters were applied for the mDSC measurements: a linear heating rate of 2 °C/min combined with a modulation amplitude of 0.212 °C and a period of 40 s. Approximately 1–3 mg of the sample were accurately weighed into standard aluminum DSC pans (TA Instruments, Zellik, Belgium), followed by crimping with standard aluminum DSC lids (TA Instruments, Zellik, Belgium). All samples were isothermally held at 40 °C for 30 min, followed by a heating procedure ranging from −20 to 120 °C. DSC thermograms were analyzed using Trios software (Version 5.1, TA Instruments, Leatherhead, UK). Glass transition temperatures (T_g_) were measured at half height of transition in the reversing heat flow (RHF).

#### 2.6.2. X-ray Powder Diffraction (XRPD)

XRPD was performed using an X’Pert PRO diffractometer (PANalytical, Almelo, The Netherlands) with a Cu tube (Kα λ = 1.5418 Å) and a generator set at 45 kV and 40 mA. Measurements were executed at room temperature in transmission mode, using Kapton^®^ Polyimide Thin-films (PANalytical, Almelo, The Netherlands). The following experimental settings were selected: continuous scan mode from 4° to 40° 2θ with 0.0167° step size and 400 s counting time. Diffractograms were analyzed using X’Pert Data Viewer (Version 1.9a, PANalytical, Almelo, The Netherlands).

### 2.7. Scanning Electron Microscopy (SEM)

SEM was performed on pure MCC beads and on pure MCC tablets to select the right compression force based on the visual observed surface porosity. This technique was also used to evaluate the thickness of the surface coating on tablets and on beads. Samples were adhered to SEM stubs using double-sided carbon tape (Ted Pella Inc., California, CA, USA) and were platinum coated under vacuum with a SCD-030 Balzers Union sputter-coater (Oerlikon Balzers, Balzers, Liechtenstein). A Philips XL30 SEM-FEG (Philips, Eindhoven, The Netherlands), equipped with a Schottky field emission electron gun and a conventional Everhart-Thornley secondary electron detector, was used to record the images.

### 2.8. Thermogravimetric Analysis (TGA)

Residual solvent levels were determined with a thermogravimetric analyzer Q500 (TA Instruments, Leatherhead, UK) for coated beads and tablets’ surfaces. The same sample preparations as for mDSC measurements were carried out. The samples (approximately 3–4 mg) were heated at 10 °C/min to 100 °C in ambient atmosphere and held isothermally for 10 min. The TGA curves were analyzed using the Universal Analysis software (Version 5.5, TA Instruments, Leatherhead, UK).

## 3. Results

### 3.1. Drug Selection

**Figure 1:**
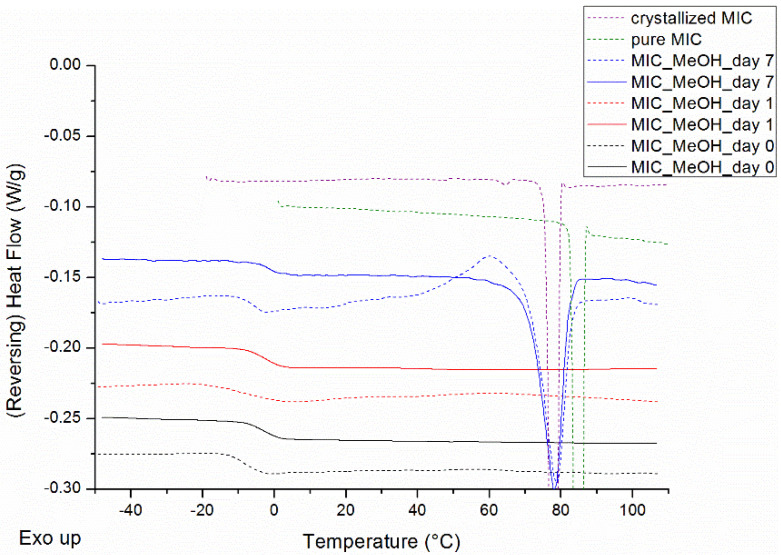
Modulated differential scanning calorimetry (mDSC) thermograms of spray dried miconazole (MIC) in methanol (MeOH), right after spray drying (black), after 1 day (red) and after 7 days (blue) of storage, and the mDSC thermogram of pure MIC (green), i.e., polymorph I, and the mDSC thermogram of MIC crystallized from a saturated MeOH solution (purple) as comparison. Reversing heat flow (RHF) signals are represented by solid lines and total heat flow (THF) signals by dashed lines. RHF and THF signals are both shown as arbitrary units.

MIC was evaluated for its low crystallization tendency in MeOH. The mDSC thermograms of Figure 1 demonstrate that the drug can be made completely amorphous by spray drying a solution of the pure drug in MeOH. After only one week, a melting endotherm appears, signaling crystallinity. Notably, at this timepoint, part of the amorphous drug was prone to crystallization because of the heating procedure during the mDSC measurement, and this crystallization event can be clearly seen in the total heat flow (THF) of Figure 1 (dashed blue curve). It is, however, striking that the melting point onsets (T_m,average_ = 72.8 °C), determined in the THF, are lower than the melting point onsets of any known MIC polymorph (polymorph I, i.e., thermodynamically most stable polymorph, T_m_ = 83.6 °C (see dashed green curve of Figure 1) and polymorph II T_m_ = 78.8 °C (not shown)) [19]. Therefore, MIC was crystallized from a saturated MeOH solution and the resulting crystals were analyzed with mDSC. The resulting melting points (T_m,average_ = 76.4 °C) do in this case correspond to the MIC melting points obtained after spray drying, as visualized in Figure 1. Hence, the melting enthalpy of MIC, crystallized from a saturated MeOH solution, was used as a reference for crystallinity percentage calculations. Taking into account the crystallization event, the average crystallinity percentage of spray-dried MIC after one week of storage amounts to 16.6%. With respect to the classification of Van Eerdenbrugh et al., it can thus be concluded that MIC also exhibits a low crystallization tendency in MeOH [20]. Despite the melting endotherm, observed with mDSC after one week, crystallinity could not be detected with XRPD and hence it cannot be decided which polymorphic form of MIC has been formed. An additional small melting endotherm around 62 °C could be observed in the mDSC thermograms of MIC, crystallized from a saturated MeOH solution, thereby suggesting the formation of a MIC solvate.

### 3.2. Determination of the Highest Possible Drug Loading: Film Casting and Bead Coating

A drug loading screening was performed on casted films and coated beads in order to define the highest possible MIC loading that still results in a one phase amorphous system. A MIC load (i.e., drug weight fraction) of 30% was applied as starting point and drug loadings were further increased with consecutive steps of 5%.

#### 3.2.1. Film Casting

**Figure 2:**
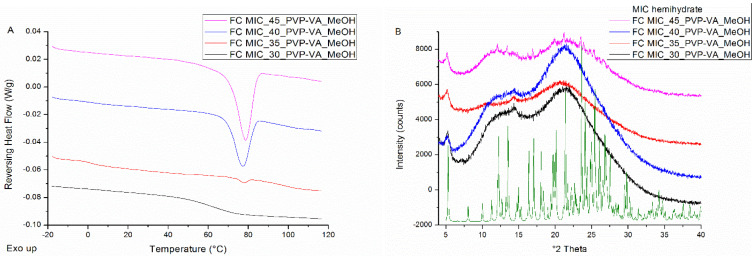
(**A**) mDSC thermograms of film casted (FC) MIC-poly(vinylpyrrolidone-co-vinyl acetate) (PVP-VA) systems, increasing the drug loading from 30% (black) to 45% (pink) by intermediate steps of 5%. The RHF signals are shown as arbitrary units; (**B**) corresponding X-ray powder diffraction (XRPD) diffractograms and the diffractogram of MIC hemihydrate (green) as comparison. The intensities are shown as arbitrary units. The small observable peaks just before 15° are due to the Kapton^®^ Polyimide Thin-films since XRPD measurements were conducted in transmission mode.

The mDSC thermograms of film casted MIC-PVP-VA systems with 30% drug loading show a single T_g_ and hence indicate the formation of a one phase amorphous system (see Figure 2A). When the drug loading is increased, melting events appear from 35% drug loading on and point out the presence of crystalline content. In addition, for MIC-35-PVP-VA (i.e., the formulation with 35% drug loading), the T_g_ around 1.5 °C reveals the occurrence of amorphous demixing (T_g_(MIC) = 1.7 °C) [19]. Note that the T_g_ of the drug-polymer system could, however, not be detected from 35% drug loading on, which may be attributed to the melting transitions and/or the inhomogeneity of the casted films. More detailed information regarding the mDSC thermograms of film casted MIC-PVP-VA systems can be found in Table 1. The corresponding XRPD diffractograms only show distinct Bragg peaks for the 45% drug loading systems and in accordance with the diffractogram of the MIC hemihydrate, as depicted in Figure 2B. The presence of an amorphous halo for the 35% and 40% drug loading systems, despite the melting events on their corresponding thermograms, can be explained by the higher sensitivity of mDSC compared to XRPD [21]. It is thus hypothesized that too few crystals are formed over the entire surface of the films to be detected with XRPD or that the length of coherence is reduced.

#### 3.2.2. Bead Coating

**Figure 3:**
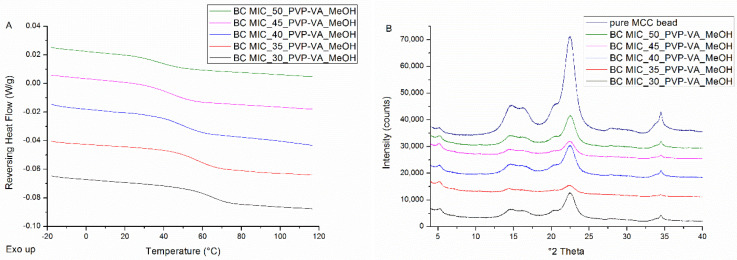
(**A**) mDSC thermograms of milled MIC-PVP-VA coated beads (BC), with drug loadings from 30% (black) to 50% (green) by intermediate steps of 5%. The RHF signals are shown as arbitrary units; (**B**) corresponding XRPD diffractograms and the diffractogram of pure microcrystalline cellulose (MCC) beads (dark blue) as comparison. The intensities are shown as arbitrary units.

All solid dispersions coated on beads were characterized by single T_g_s (see Figure 3A), indicating the formation of one phase amorphous systems, and by the absence of distinct Bragg peaks (see Figure 3B). The peaks that can be observed on the diffractograms are attributable to the semi-crystalline nature of MCC beads. As can be derived from Figure 3A and from the numerical values reported in Table 2, systems are characterized by lower T_g_ values and broader T_g_ widths as drug loading increases.

### 3.3. Development of a Surface Coating Technique

**Figure 4:**
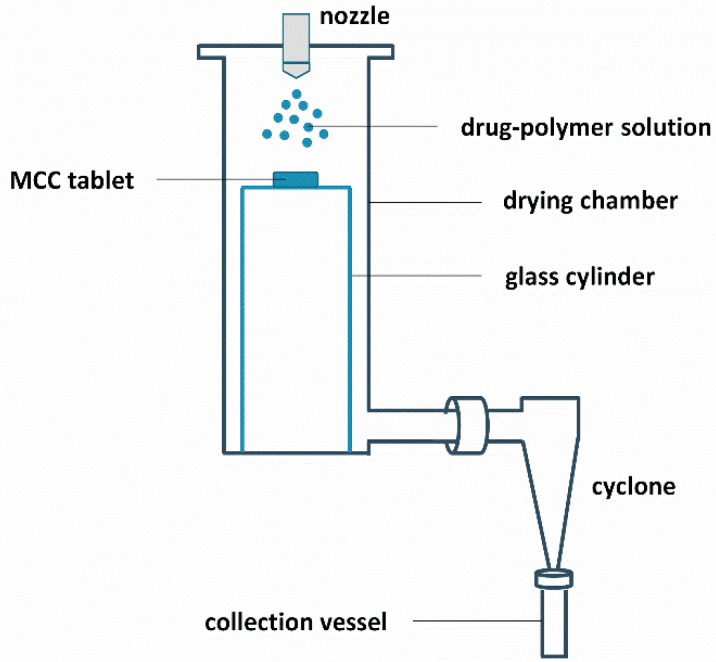
Schematic illustration of the surface coating setup.

The results above indicate that film casting is not suitable for phase behavior predictions of ASDs manufactured with bead coating. The main aim of this research paper was therefore to investigate whether and how a surface coating technique can be developed that can predict the phase behavior of MIC-PVP-VA solid dispersions coated on beads. For this purpose, the drying chamber of the spray dryer was flattened at the bottom so that a glass cylinder, with a diameter of 9 cm and height of 28 cm, could be inserted. This cylinder serves as supporting plate for an MCC tablet and its dimensions were chosen in such a way that the setup still allows for sufficient air flow for the simultaneous production of spray dried powder that can be collected in the collection vessel. The drying air temperature in the drying chamber was set at 40 °C, which corresponds to the T_bed_ during the bead coating procedure, and the drying air flow rate was set at 33 m³/h. The feed solution flow rate was set at 7 mL/min, based on visual assessments of the coating, since lower feed rates resulted in the appearance of spray dried powder on the tablet surface. For the atomization of the drug-polymer solution, a bi-fluid nozzle (2.8 mm diameter) was used and an atomization air flow rate of 10 L/min was applied. When the coating procedure was finished, an additional drying step of 5 min was implemented, which corresponds to the drying step at the end of the bead coating procedure. A schematic illustration of the surface coating setup is provided in Figure 4.

#### 3.3.1. Compression Force

**Figure 5:**
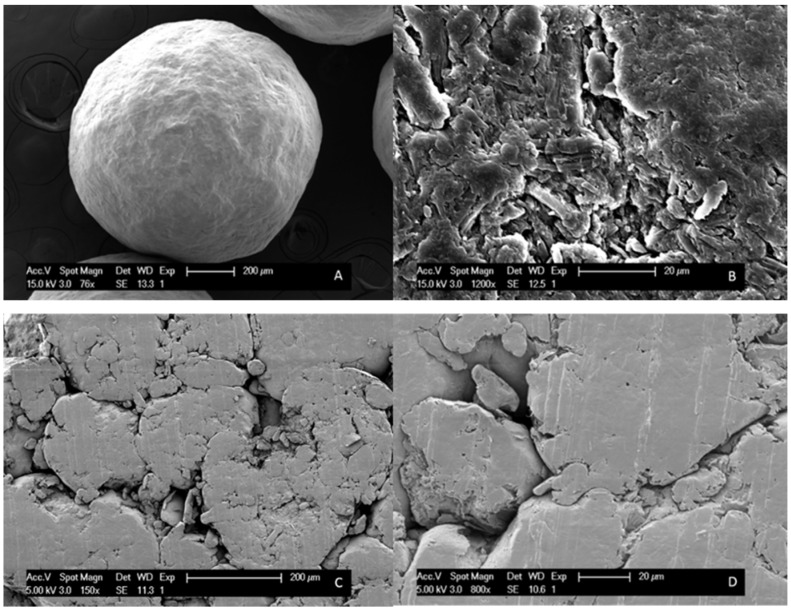
SEM images of (**A**) an MCC bead with 76× magnification; (**B**) an MCC bead with 1200 × magnification. SEM images of (**C**) an MCC tablet prepared with 184.8 MPa compression force; (**D**) an MCC tablet prepared with 369.5 MPa compression force.

MCC tablets were obtained after milling MCC beads, and the sieving and compression of the resulting MCC powder. We attempted to attain a surface porosity comparable to that of a pure MCC bead and hence a compression force of 369.5 MPa was selected, based on the generated SEM images (see Figure 5).

#### 3.3.2. Spraying Procedure

**Figure 6:**
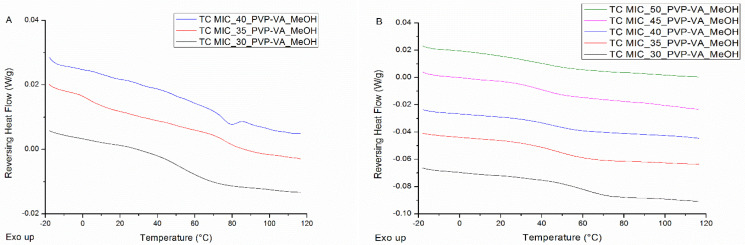
mDSC thermograms of (**A**) MCC tablets surface coated (TC) with MIC-PVP-VA, applying the continuous spraying procedure; (**B**) MCC tablets surface coated with MIC-PVP-VA, employing the intermittent spraying procedure IM1 (20 s–20 s). The RHF signals are shown as arbitrary units.

When coating the surface of an MCC tablet, either a continuous or an intermittent spraying procedure can be applied. With the former procedure, 20.0 mL of MIC-PVP-VA_MeOH solution is continuously sprayed onto the tablet’s surface, followed by an additional drying step of 5 min. The phase behavior of the surface coated ASDs, when applying this continuous spraying procedure, is depicted in Figure 6A. For the MIC-PVP-VA systems with 30% drug loading, one T_g_ is obtained, implying the formation of a one-phase amorphous system. For the formulations with 35% drug loading, mDSC thermograms indicate amorphous demixing, i.e., a drug-rich phase (T_g,average_ = 5.0 °C) and a polymer-rich phase (T_g,average_ = 76.8 °C). Moreover, in two out of three batches, an additional small melting endotherm could be detected in the THF (data not shown). If the drug loading is further increased to 40%, either one-phase amorphous systems were produced (data not shown) or formulations containing crystalline content were obtained (two out of three batches). This phase behavior inconsistency can be ascribed to the highly variable character of the continuous spraying procedure, involving the lack of adequate kinetic trapping. In contrast, when an intermittent spraying procedure is employed, spraying alternates with drying, followed by an additional drying step of 5 min at the end. Two distinct intermittent procedures were evaluated: one where 20 s of spraying alternates with 20 s of drying (denoted as IM1), and one where 20 s of spraying alternates with 40 s of drying (denoted as IM2). The mDSC thermograms of Figure 6B illustrate that, with the IM1 procedure, one-phase amorphous systems are produced up to 50% drug loading. Similar results were acquired for the IM2 procedure and are reported in more detail in Table 3. Note that these high drug-loaded ASDs may be oversaturated and could thus be more prone to destabilization on the long term. It can be clearly seen that average T_g_ values decrease with increasing MIC weight fraction. Despite the longer drying period in between spraying steps for IM2, the obtained T_g_ values are comparable to the T_g_ values for IM1. The XRPD diffractograms of all surface coated tablets displayed an amorphous halo (data not shown), and hence mDSC was found to be decisive in the development of the surface coating technique and more specifically, in the distinction between the intermittent and continuous spraying procedure.

#### 3.3.3. Comparison to Bead Coating

**Figure 7:**
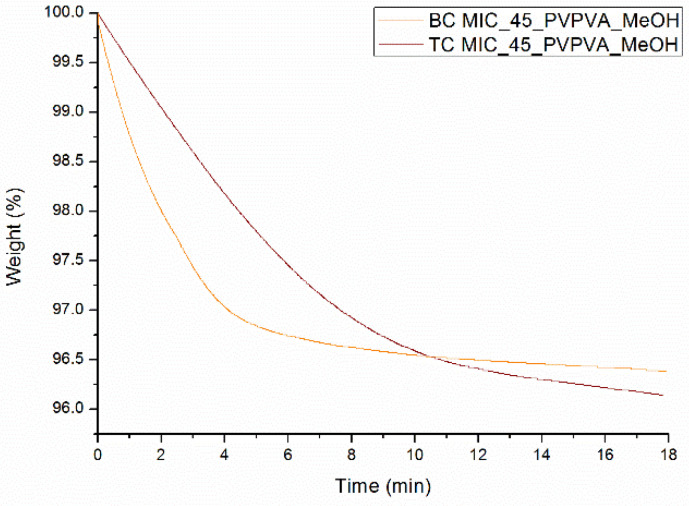
TGA curves of MIC_45_PVP-VA_MeOH systems prepared with bead coating (BC) and surface coating (TC).

From the results reported above, it can be concluded that the newly developed surface coating technique can predict the phase behavior of MIC-PVP-VA ASDs coated on beads, but only if an intermittent spraying procedure is applied. Nevertheless, when comparing the mDSC thermograms of Figure 3A and Figure 6B, and their more detailed information reported in Table 2 and Table 3, some differences between bead coating and surface coating can be noticed. First, lower T_g_ values are generally obtained for the surface coating technique, and it was assumed that more residual solvent was present in the scraped off tablet coating flakes, as compared to coated beads. To test this hypothesis, TGA was conducted, which allows the determination of the residual solvent level by recording weight loss (due to solvent evaporation) as a function of time. The resulting TGA curves are depicted in Figure 7 and show that the solvent evaporates slower from the scraped off tablet coating flakes than from the milled coated beads. The tablet coating flakes and milled coated beads are characterized by a weight loss of 3.9% and 3.5%, respectively, indicating that more residual solvent is present when using the surface coating technique, thereby confirming the aforementioned hypothesis.

**Figure 8:**
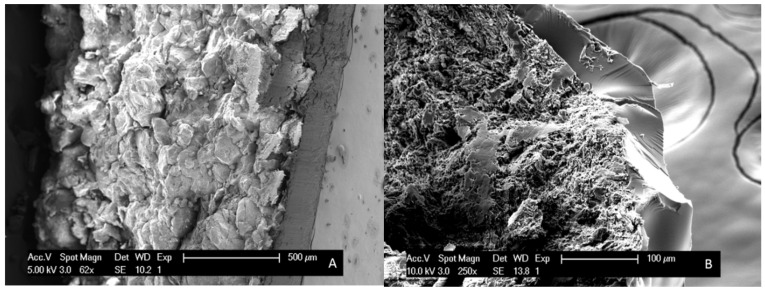
SEM images of (**A**) a MIC_45_PVP-VA_MeOH surface coated MCC tablet, employing the intermittent spraying procedure IM1; (**B**) a MIC_45_PVP-VA_MeOH coated MCC bead.

A second difference that could be identified between bead coating and surface coating is the larger T_g_ widths (i.e., broader T_g_s) for the latter, which implies that more heterogeneous systems are produced (see Table 2 and Table 3). The coated tablets could thus potentially lead to destabilization in the long term. Furthermore, variations in coating thickness were perceived by means of SEM images and are illustrated in Figure 8. The coating thickness of MIC_45_PVP-VA solid dispersions on tablets, thus resulting from the newly developed surface coating technique, is approximately 150 µm, whilst thinner coatings (±60 µm) were obtained for MIC_45_PVP-VA solid dispersions coated on beads.

#### 3.3.4. Repeatability

Repeatability of the surface coating technique with the intermittent spraying procedure was assessed for MIC-PVP-VA systems with 35 and 45% drug loading. Both intermittent procedures, IM1 and IM2, were evaluated and the results are reported in Table 4. The same phase behavior and, more importantly, comparable T_g_ values were obtained for the three batches of all samples studied, demonstrating the repeatability of the newly developed surface coating technique. The small observable differences in T_g_ values are most probably attributable to distinct residual solvent levels between the samples.

### 3.4. Determination of the Highest Possible Drug Loading: Spray Drying

**Figure 9:**
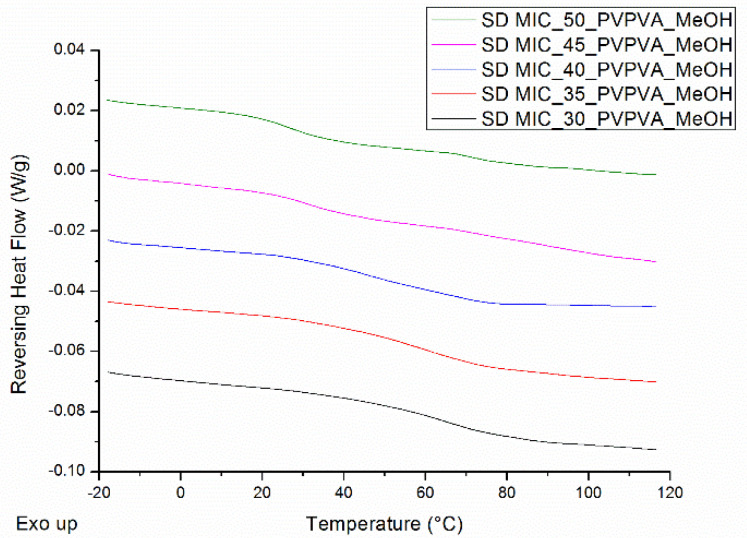
mDSC thermograms of spray dried (SD) MIC-PVP-VA systems, with drug loadings from 30% (black) to 50% (green) by intermediate steps of 5%. The RHF signals are shown as arbitrary units.

The mDSC thermograms of spray dried MIC-PVP-VA systems indicate the formation of one-phase amorphous systems for the formulations with 30%, 35% and 40% MIC weight fraction (see Figure 9). When the drug loading is increased, two T_g_s appear, implying the generation of amorphous–amorphous phase separated systems. For the spray-dried powders, only mDSC thermograms are described, since XRPD showed an amorphous halo for all drug loadings.

Note that the spray dried material was produced simultaneously with the ASD coated tablets, as illustrated in Figure 4. The surface coating technique was therefore also evaluated for its repeatability regarding the production of spray dried powder for MIC-PVP-VA systems with 35 and 45% drug loading. The results are reported in Table 5 and indicate the formation of one-phase amorphous systems and comparable T_g_ values for all batches of MIC_35_PVP-VA, demonstrating the repeatability of the developed surface coating technique for the production of spray dried powder. For the formulations with 45% drug loading, the outcomes depend on the applied spraying procedure. In the case of IM1, the formation of amorphous–amorphous phase separated systems was repeatable. In contrast, when IM2 was used, the polymer-rich phase could not always be detected with mDSC, suggesting that 45% drug loading defines the boundary between one-phase amorphous systems and amorphous demixing.

## 4. Discussion

MIC is classified in the literature as a compound that remains completely amorphous or that is merely slightly crystalline one week after preparation, according to the semi-quantitative classification of Van Eerdenbrugh et al. [20]. However, since the employed solvent system can have an influence on the classification outcome, and the crystallization tendency of MIC was only reported for dichloromethane (DCM) and ethanol (EtOH), MIC was evaluated for its crystallization tendency in MeOH. One should keep in mind that, as well as the solvent, the classification also depends on the manufacturing method and the storage conditions applied. It was found that MIC also exhibits a low crystallization tendency in MeOH. Nevertheless, crystalline content could not be detected with XRPD and thus it could not be concluded which polymorphic form of MIC has been formed. The XRPD diffractograms of MIC, crystallized from a saturated MeOH solution, do not correspond to any MIC polymorph that is currently reported in the literature [19,22]. The additional small melting endotherm around 62 °C, that could be observed on the mDSC thermograms of MIC, crystallized from a saturated MeOH solution, suggests the formation of a MIC solvate. A possible MIC solvate formation can be founded by the reported MIC hemihydrate structure and by the publication of Kersten et al., who reported a hemi-hydrogen peroxide (HP) solvate of MIC [22,23]. In both cases, one solvent molecule is incorporated for every two MIC molecules, via hydrogen bonding of the solvent molecule with the imidazole nitrogen atoms of MIC [22,23]. Further investigations concerning the possible formation of a new MIC solvate, encompassing the implementation of single crystal analysis, are currently ongoing.

Film casting is often used as a screening test to assess drug-polymer miscibility; however, our results indicate that this technique is not suitable for phase behavior predictions of ASDs manufactured with spray drying or bead coating, because of the low rate of solvent evaporation. The main aim of this research paper was therefore to investigate whether and how a surface coating technique can be developed that can predict the phase behavior of MIC-PVP-VA ASDs coated on beads. A surface coating setup was established inside the drying chamber of a spray dryer and hence allows the simultaneous and non-interfering production of spray dried powder. The operational conditions were related as closely as possible to the parameter settings of the bead coating process. Optimal process parameters of the latter were selected based on preliminary trials since, to the best of our knowledge, no references could be found concerning fluid bed coating of MIC. Overall, it can be concluded that the newly developed surface coating technique can predict the phase behavior of MIC-PVP-VA ASDs coated on beads, but only if an intermittent spraying procedure is applied. Indeed, this intermittent spraying resembles the layering approach of the bead coating process. Contrarily, the continuous spraying procedure was found to be highly variable due to a lack of kinetic trapping and the resulting phase behavior was similar to what was obtained from film casting experiments. Despite the fact that the predictive value and the repeatability of the intermittent surface coating technique is demonstrated, further optimizations to lower residual solvent levels of the coated tablets are of interest. Furthermore, it should also be examined in the future whether this technique is also predictive for a fast crystallizing model drug. In this case, it will be of utmost importance to align the coating thickness of the tablets to the one of the beads and to evaluate surface crystallization.

In addition to the development of the surface coating technique and the assessment of its predictive value, the technique was also evaluated for its ability to manufacture high drug-loaded ASDs, in comparison to bead coating, spray drying and film casting. In ASDs, relatively low drug loadings are generally utilized (ca. 30–35%), considering potential physical stability issues. It is, however, indispensable to obtain drug loadings as high as possible, aiming to lower the pill burden and/or reduce the dosage size to enhance the therapeutic compliance of the patient [24]. Nevertheless, the reader should keep in mind that the high drug-loaded ASDs may be oversaturated and could thus be more prone to destabilization in the long term, which underpins the necessity of physical stability studies. The comparative evaluation of these solvent-based manufacturing techniques contributes to a better understanding of the bead coating process and its value in relation to spray drying. For the film casted MIC-PVP-VA formulations, 30% was determined as the highest possible drug loading that still results in a one-phase amorphous system, since a melting point appeared on the mDSC thermograms for the formulations with 35% drug loading (see Figure 2A). Film casting thus performed the worst in obtaining high drug-loaded ASDs, as compared to the other solvent-based ASD manufacturing techniques. This can be explained by the fact that the solvent evaporation is extremely slow, thereby entailing the absence of kinetic trapping of the physical structure of the drug–polymer system. XRPD measurements of the casted films indicated that MIC crystallized into its hemihydrate form. Since, to the best of our knowledge, no thermogram of the pure MIC hemihydrate is reported, crystallinity percentages could not be calculated. The discrepancy in phase behavior between the mDSC thermograms and the corresponding XRPD diffractograms for casted films underpins the importance of implementing complementary solid-state analytical techniques to provide a comprehensive characterization of the prepared ASDs [21]. For MIC-PVP-VA formulations coated on beads, even 50% drug loading resulted in the formation of one-phase amorphous systems. However, the T_g_ widths became broader as the MIC loading increased, indicating that the one-phase amorphous systems became more heterogeneous (see Table 2). It should be noted that the formulation with 50% drug loading was coated onto MCC beads with other parameter settings. If the same parameter settings as for the other drug loadings were applied, agglomeration problems were encountered. This can be explained by the fact that for the drug-polymer system with 50% drug loading, the T_g_ (T_g,average_ = 36.4 °C) fell below the T_bed_ (± 40 °C), resulting in stickiness. Hence, in order to manufacture this formulation, the T_inlet_ and feed rate were lowered. For MIC-PVP-VA formulations coated on tablets, the 50% drug loading also resulted in the formation of one-phase amorphous systems, explaining the predictive power of the intermittent surface coating procedure. Contrarily, the ASDs coated on the MCC tablet surface are characterized by the highest possible drug loading of 30% if the continuous spraying procedure is applied, and this is hence similar to what was obtained from film casting experiments. For the spray-dried MIC-PVP-VA systems, 40% was determined as the highest possible drug loading that still results in a one-phase amorphous system, because of the occurrence of amorphous demixing for higher drug loadings.

## 5. Conclusions

This study investigated whether and how a technique could be developed that can predict the phase behavior of ASDs coated on beads. For this purpose, MIC-PVP-VA in MeOH was selected as the model system. A surface coating setup was established inside the drying chamber of a spray dryer, that simultaneously allows the coating of a tablet surface and production of spray dried powder. This newly developed surface coating technique was able to predict the phase behavior of MIC-PVP-VA ASDs coated on beads, when an intermittent spraying procedure was applied. The technique was also evaluated for its ability to manufacture high drug-loaded ASDs, in comparison to bead coating, spray drying and film casting. The highest possible drug loadings that still resulted in one-phase amorphous systems were 30% for film casting and surface coating with a continuous spraying procedure and 40% for spray drying. ASDs obtained with bead coating and with surface coating with an intermittent spraying procedure were still one-phase amorphous for 50% drug loading. Note that this ranking is only valid just after production and hence, physical stability studies are imperative for future research. Nevertheless, these results already give an indication of the potential of the underexplored ASD manufacturing technique and encourage further studies concerning this topic. The newly developed surface coating technique forms an important first step towards a better understanding of the bead coating process and its value in relation to spray drying. Eventually, surface coating could even serve as a screening tool and could offer interesting opportunities to select the right drug-polymer candidates for bead coating purposes within the pharmaceutical industry.

## Figures and Tables

**Table 1:** Average T_g_ values and widths ± standard deviation (sd) for MIC_PVP-VA_MeOH films. Average T_m,onset_ values and melting enthalpies ± sd are reported as well.

Drug Weight Fraction (%)	Average Tg Value ± Sd (°C)	Average Tg Width ± Sd (°C)	Average Tm,onset ± Sd (°C)	Average Melting Enthalpy ± Sd (J/g)
30	62.4 ± 0.8	23.7 ± 4.7	/	/
35	1.7 ± 0.9 (*)	7.6 ± 1.6	75.7 ± 0.6	1.1 ± 0.2
40	/	/	75.1 ± 1.2	3.6 ± 2.0
45	/	/	69.7 ± 2.9	18.0 ± 4.6

(*) The T_g_ of the drug-polymer system could not be detected.

**Table 2:** Average T_g_ values and widths ± standard deviation (sd) for MIC_PVP-VA_MeOH coated beads. Average T_m,onset_ values and melting enthalpies ± sd do not apply for the coated beads, since no crystallinity could be detected for any formulation.

Drug Weight Fraction (%)	Average T_g_ Value ± Sd (°C)	Average T_g_ Width ± Sd (°C)
30	65.3 ± 0.9	14.1 ± 0.6
35	57.2 ± 1.6	14.7 ± 1.0
40	49.6 ± 0.8	16.7 ± 3.3
45	43.8 ± 1.0	18.5 ± 1.1
50	36.4 ± 0.3	17.9 ± 1.4

**Table 3:** Average T_g_ values and widths ± sd for MIC-PVP-VA surface coated tablets, using the intermittent spraying procedures IM1 (20 s–20 s) and IM 2 (20 s–40 s).

Drug Weight Fraction (%)	Average T_g_ Value ± Sd (°C) IM 1	Average T_g_ Width ± Sd (°C) IM 1	Average T_g_ Value ± Sd (°C) IM 2	Average T_g_ Width ± Sd (°C) IM 2
30	60.1 ± 2.5	18.4 ± 3.8	60.0 ± 1.7	15.8 ± 1.6
35	50.5 ± 0.9	23.1 ± 3.3	47.8 ± 0.5	24.7 ± 2.8
40	45.0 ± 0.4	17.7 ± 1.0	45.5 ± 1.5	16.1 ± 2.7
45	37.0 ± 1.1	19.8 ± 0.7	35.9 ± 2.2	16.8 ± 8.0
50	31.8 ± 5.1	30.4 ± 7.6	34.1 ± 0.3	17.9 ± 0.8

**Table 4:** Repeatability of the surface coating procedure. Average T_g_ values ± sd for MIC_PVP-VA surface coated tablets, using the intermittent spraying procedures IM1 (20 s–20 s) and IM 2 (20 s–40 s).

Sample	Average T_g_ Value ± Sd (°C) Batch 1	Average T_g_ Value ± Sd (°C) Batch 2	Average T_g_ Value ± Sd (°C) Batch 3
TC_MIC_35_PVP-VA_MeOH_IM1	50.5 ± 0.9	54.1 ± 0.7	49.1 ± 6.1
TC_MIC_35_PVP-VA_MeOH_IM2	47.8 ± 0.5	53.3 ± 1.2	55.3 ± 1.0
TC_MIC_45_PVP-VA_MeOH_IM1	37.0 ± 1.1	33.1 ± 4.4	37.5 ± 3.1
TC_MIC_45_PVP-VA_MeOH_IM2	35.9 ± 2.2	35.4 ± 1.6	38.4 ± 2.5

**Table 5:** Repeatability of the surface coating procedure. Average T_g_ values ± sd for MIC-PVP-VA spray dried powder, using the intermittent spraying procedures IM1 (20 s–20 s) and IM 2 (20 s–40 s). Two T_g_ values ± sd are reported for amorphous-amorphous phase separated systems.

Sample	Average T_g_ Value(s) ± Sd (°C) Batch 1	Average T_g_ Value(s) ± Sd (°C) Batch 2	Average T_g_ Value(s) ± Sd (°C) Batch 3
SD_MIC_35_PVP-VA_MeOH_IM1	52.6 ± 4.7	51.6 ± 0.4	51.2 ± 3.2
SD_MIC_35_PVP-VA_MeOH_IM2	52.5 ± 4.6	50.0 ± 1.3	49.6 ± 2.5
SD_MIC_45_PVP-VA_MeOH_IM1	34.7 ± 3.3 71.4 ± 0.5	36.1 ± 0.6 71.8 ± 1.6	36.9 ± 1.9 69.9 ± 2.6
SD_MIC_45_PVP-VA_MeOH_IM2	35.5 ± 3.7 71.7 ± 3.4	37.6 ± 0.9 (*)	37.4 ± 0.6

(*) The mDSC thermograms of the spray dried powder showed amorphous demixing in only one out of three batches.
